# Retinal Nerve Fibre Layer and Macular Thicknesses in Adults with Hyperopic Anisometropic Amblyopia

**DOI:** 10.1155/2015/946467

**Published:** 2015-05-07

**Authors:** Konuralp Yakar, Emrah Kan, Aydın Alan, Mehmet Hanifi Alp, Tolga Ceylan

**Affiliations:** ^1^Ophthalmology Department, Ataturk State Hospital, 57000 Sinop, Turkey; ^2^Ophthalmology Department, Training and Research Hospital, 55100 Samsun, Turkey; ^3^Ophthalmology Department, Palandöken State Hospital, 25080 Erzurum, Turkey; ^4^Ophthalmology Department, Trakya University Hospital, 22030 Edirne, Turkey

## Abstract

*Objectives*. This study compared the macular and retinal nerve fibre layer (RNFL) thicknesses and optic nerves of eyes with reduced vision due to anisometropia with the contralateral healthy eyes in adults using optical coherence tomography (OCT). *Methods*. This cross-sectional study was conducted in Atatürk State Hospital, Sinop, Turkey. Macular and RNFL thicknesses, optic nerve disc area, cup area, and horizontal and vertical cup-to-disc ratios obtained using a NIDEK RS-3000 SLO spectral domain OCT device were compared between the amblyopic and fellow eyes in 30 adults with anisometropic amblyopia 18–55 years old who were seen in our clinic with unilateral poor vision. *Results*. The mean macular thickness was 266.90 ± 23.22 *µ*m in the amblyopic eyes and 263.90 ± 22.84 *µ*m in the fellow eyes, and the mean RNFL thickness was 111.90 ± 12.9 and 109.70 ± 9.42 *µ*m, respectively. The two thicknesses did not differ significantly between the amblyopic and fellow eyes. There were also no significant differences between the eyes in disc area, cup area, and horizontal-vertical cup/disc ratios. *Conclusion*. There does not seem to be a difference in macular thickness, peripapillary RNFL, or optic disc structures between the amblyopic and fellow eyes in adults.

## 1. Introduction

Amblyopia is a neuroanatomical and neurophysiological ophthalmological disorder with no associated pathology of the optical axis or macula that could cause low vision; it cannot be eliminated with refractive correction and may be treated if diagnosed at an early stage. Amblyopia may be classified as strabismic, refractive (anisometropic and isometropic), deprivational, idiopathic, and mixed types [1, 2]. Anisometropia has been defined as a condition in which there is a difference of 0.5–2 dioptres in refractive error between the two eyes [3–7]. In anisometropic amblyopia, focused and unfocused images coming out of the point of fixation produce a blurred image in the fovea of the eye and an abnormal binocular interaction develops to the disadvantage of the eye with blurred vision as a result of overlapping clear and blurred images, leading to inhibition of the fovea and poor vision in that eye [2]. Studies have demonstrated that the lateral geniculate nucleus and visual cortex are the structures that are primarily affected in amblyopia [8–10].

Studies have investigated involvement of the macula and optical nerve in amblyopia and while some researchers found an increase in the retinal nerve fibre layer (RNFL) thickness or macular thickness, others did not observe any difference [11–17].

Optical coherence tomography (OCT) was first described in 1991 and first used in ophthalmology practice in 1995. It allows us to perform an optical biopsy of tissues by taking advantage of the differential optical refraction properties of different tissues and to examine 10 *μ*m thick sections. It is a noncontact, noninvasive, easily reproducible method that uses only light.

This study compares macular thickness, RNFL thickness, and optical disc parameters in the anisometropic amblyopic eyes and contralateral healthy eyes of adults using spectral domain scanning laser ophthalmoscope (SLO) OCT and investigated whether amblyopia affected these structures.

## 2. Materials and Methods

This study was approved by the Ethics Committee of Samsun 19th May University, Turkey, and complied with the tenets of the Declaration of Helsinki for research involving human tissue. Informed consent was obtained from the patients after explaining the research.

This cross-sectional study was conducted in Atatürk State Hospital, Sinop, Turkey. OCT findings were obtained from 30 adults with anisometropic amblyopia 18–55 years old who were seen in our clinic with unilateral poor vision. We defined anisometropia as a difference in spherical equivalent of at least 1.00 dioptre hypermetropia or 1.00 dioptre simple astigmatism between the two eyes. Patients with a history of diabetes mellitus, glaucoma, strabismus, cataract, a hereditary or acquired retinal or optical disc disorder, panretinal photocoagulation, intravitreal injection, nystagmus, intraocular surgery, or trauma were excluded. Myopic amblyopia was also excluded because retinal changes (Bruch membrane rupture, choroidal neovascularization, exudative or atrophic macular degeneration, etc.) in the condition could affect OCT parameters. The study included 60 eyes of 30 patients with the vision of one eye reduced by at least two lines on the Snellen chart due to hypermetropic (at least 1.00, maximum 5.00) or astigmatic (at least 1.00, maximum 3.00) dioptre refractive error compared to the other eye with full vision.

The routine ophthalmologic examination of the study subjects included the best corrected visual acuity on the Snellen chart, cycloplegic refractive error as measured with a NIDEK ARK-1 (Tokyo, Japan) autorefractometer, intraocular pressure as measured by Goldmann applanation tonometry, and biomicroscopic and fundus examinations. A detailed history was taken to identify any hereditary retinal or optic disc disorders. Macular thickness, peripapillary RNFL thickness, disc area, cup area, and horizontal and vertical cup/disc ratio were obtained with a macula map, disc map, and macula line software by the same technician using a NIDEK RS-3000 SLO spectral domain OCT (Tokyo, Japan) device for all patients. Measurements were repeated until images of ≥9/10 quality were obtained.

**Figure 1:**
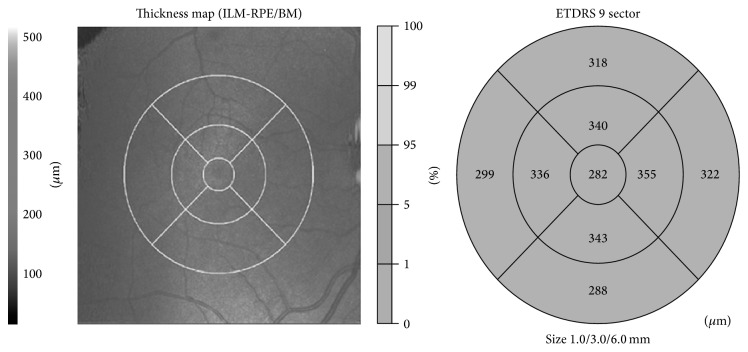
Macular thickness map diagram and ETDRS grid.

Macular thickness was evaluated in nine quadrants using the Early Treatment Diabetic Retinopathy Study (ETDRS) grid comprising three concentric circles with diameters of 1, 3, and 6 mm. The quadrants were named the central zone, inner superior-nasal-inferior-temporal, and exterior superior-nasal-inferior-temporal from innermost to outermost (Figure 1). Each area was compared with the corresponding area in the fellow eye.

**Figure 2:**
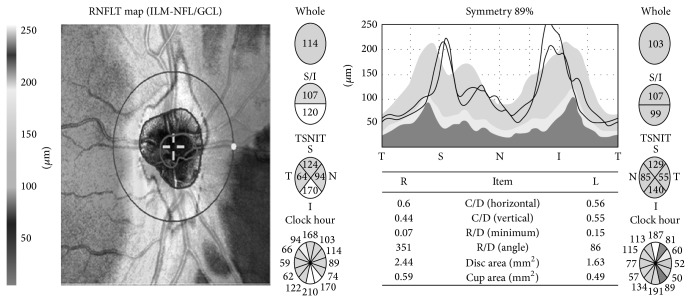
Optic disc parameters and retinal nerve fiber layer diagram.

Using disc map data for the patients, the peripapillary RNFL was compared in terms of total thickness and the thickness in each of the superior, inferior, nasal, and temporal quadrants. Disc area, cup area, and horizontal and vertical cup/disc ratios were also compared (Figure 2).

### 2.1. Statistical Analysis

All analyses were performed using SPSS for Windows, version 18.0 (SPSS, Chicago, IL). Results are presented as the means ± standard deviations (SD) for continuous data or as percentages and numbers for categorical data. Macular thickness in nine quadrants, the average thickness and that of the four quadrants of the RNFL, and optic disc parameters were compared between the two eyes of the patients using a paired *t*-test (two-tailed). The associations between refractive error and retinal OCT variables were determined using Spearman's correlation. Two-sided *P* values <0.05 were considered statistically significant.

## 3. Results

The study included 60 anisometropic amblyopic eyes of 30 patients (18 females, 12 males) with a mean age of 34.7 ± 11.83 (range: 18–55) years. Of the patients, 14 had amblyopia in their right eye and 16 in their left eye. Twenty had only hypermetropia, five had both hypermetropia and astigmatism, and five had only astigmatic refraction. The average best corrected visual acuity of the amblyopic eyes was 0.5 ± 0.12 (range: 0.05 to 0.7) on the Snellen chart. The mean spherical equivalent refractive error was +3.25 ± 0.55 (range: +1.00 to +5.00) dioptres in the hyperopic amblyopic eyes and +1.00 ± 0.25 (range: +0.50 to +1.50) dioptres in the fellow eyes. There was a significant difference in refractive error between the amblyopic and fellow eyes (paired *t*-test, *P* < 0.001). Central macular thickness was 266.90 ± 23.22 *μ*m in the amblyopic eyes and 263.90 ± 22.84 *μ*m in the fellow eyes. There was no significant difference in central macular thickness or macular thickness in the eight quadrants from central to peripheral zone between the healthy and amblyopic eyes. The average macular thicknesses obtained with the ETDRS grid in eight quadrants from innermost to outermost are summarized in Table 1. The mean total RNFL thickness was 111.90 ± 12.9 *μ*m in the amblyopic eyes and 109.70 ± 9.42 *μ*m in the fellow eyes. No significant difference in overall RNFL thickness was found or in the superior, inferior, nasal, or temporal quadrants. The optic disc parameters (disc area, cup area, and cup/disc ratio) did not differ significantly between the two eyes. All of the findings and *P* values are summarized in Table 1.

When analyzed using Spearman's correlation test, the retinal OCT parameters were not correlated with the dioptre of the refractive error (Table 2).

## 4. Discussion

Amblyopia has an incidence of 1.3–3.6% in the paediatric population [18]. Although El-Shamayleh [19] showed that the visual cortex in animals can be affected in amblyopia, advances in imaging methods and the introduction of OCT into clinical practice led to renewed attention to the retina and optic nerve in amblyopia, and the involvement of these structures was investigated extensively. Conflicting results were reported for different types of amblyopia [11–17, 20–29].

In 14 unilateral hyperopic anisometropic children from 5 to 10 years of age, Wang and Taranath [20] found no significant difference in central macular thickness, total macular volume, or RNFL thickness between both eyes of the subjects. In two groups of 15 patients each with strabismic or refractive amblyopia, Dickmann et al. [21] reported a significant increase only in the macular thickness in the strabismic eyes but no significant difference between the two eyes in macular thickness, macular volume, or RNFL in the refractive amblyopic group. Xu et al. [22] failed to find a significant difference in foveal or RNFL thickness in 21 children with unilateral esotropic amblyopia. Similarly, Tugcu et al. [23] did not find a significant difference in the foveal volume, macular volume, or RNFL values between 14 persistent and 18 resolved amblyopia patients with strabismic, ametropic, and anisometropic amblyopia, aged 8 to 14 years. Using a NIDEK RS3000 OCT device in 19 anisometropic and 17 strabismic amblyopic children, Firat et al. [24] did not demonstrate a significant difference in the macular thickness, total RNFL, or RNFL values obtained in four quadrants compared to the fellow eyes and age-matched controls. Using a different approach from the aforementioned studies, Miki et al. [25] compared the RNFL of persistently amblyopic eyes with those of treated amblyopic eyes and also found no significant difference among these patients.

Contrasting these studies, Andalib et al. [14] investigated macular and RNFL thickness in 50 anisometropic and strabismic amblyopic patients 6–18 years old. In the anisometropic group, the mean macular thickness was increased significantly in the amblyopic eyes versus the fellow eyes, while there was no significant difference in the peripapillary nerve fibre layers. There was no significant correlation in these measurements in the strabismic group. In addition, Al-Haddad et al. [15] reported a significantly greater mean foveal volume in 45 patients with a mean age of 24.8 years with both anisometropic amblyopia and strabismic amblyopia. Yalcin and Balci [26] reported foveal thickening in amblyopic eyes using time-domain OCT in patients 8–14 years of age who had hypermetropic anisometropic amblyopia versus normal subjects, but no difference was found in RNFL.

In 14 paediatric patients with unilateral deprivation amblyopia, Kim et al. [27] compared the amblyopic eyes with both the contralateral healthy eyes of the patients and healthy eyes of an age-matched control group and did not find a significant difference in macular thickness among these three groups of eyes, while the RNFL was significantly thicker in the nasal quadrant in the amblyopic eyes compared to the other two groups. This was the first study to investigate macular and RNFL thickness in deprivation amblyopia.

We found no difference among the four quadrants of RNFL thickness, macular thickness, or optic disc structure in anisometropic amblyopic eyes and fellow eyes in an adult population. Walker et al. [28] found no significant difference in macular thickness or peripapillary RNFL thickness in patients with strabismic and anisometropic amblyopia in an adult patient population similar to ours. Kantarci et al. [29] compared choroidal thickness and central macular and peripapillary RNFL thickness in adults with anisometropic amblyopia and also failed to find a difference in RNFL and central macular thicknesses, in agreement with our findings.

Our study also compared optic disc structures (disc area, cup area, and horizontal-vertical cup/disc ratios) but failed to find a significant difference between amblyopic and fellow eyes. These results counter the data of Araki et al. [30], who found a significantly larger rim area and smaller cup/disc ratios (average, vertical, and horizontal) in amblyopic eyes. This might have been because their study population included strabismic, anisometropic, and mixed-type amblyopic eyes.

Our study supports Firat et al., [24] who used the same OCT device to examine a paediatric population in the same ethnic group. Our findings suggest that when the patients reach adulthood, there is no retinal remodeling that affects OCT parameters. We believe that this is why amblyopia can be treated until 12 years of age.

The retinal changes in amblyopic eyes have not yet been elucidated. The majority of previous studies examined paediatric populations. We believe that our study makes an addition to the literature, examining hyperopic amblyopic adults. The results of previous studies are still confusing because of differences in study design, OCT devices, and the subjects' race, age, and amblyopia types.

Limitations of our study were the lack of a control group including a normal population and axial length. The small sample size limited the power of the study. However, the number of patients was similar to previous studies.

In conclusion, several levels of the visual pathways and posterior segment of the eye might be or not be affected in different types of amblyopia. Further studies, including histological sections, with greater numbers of patients are required to confirm these findings.

## Figures and Tables

**Table 1:** Comparison of macular thickness, RNFL, and optic disc parameters.

	Amblyopic eye	Fellow eye	*P* value
Macular thickness			
Central zone	266.90 ± 23.22	263.90 ± 22.84	0.342
Inner superior	342.75 ± 16.89	342.90 ± 14.86	0.428
Inner nasal	346.20 ± 16.70	346.20 ± 14.02	0.516
Inner inferior	343.85 ± 13.87	340.00 ± 19.72	0.455
Inner temporal	325.65 ± 14.93	325.70 ± 15.17	0.817
Exterior superior	313.60 ± 13.64	309.75 ± 13.68	0.631
Exterior nasal	318.05 ± 17.80	318.75 ± 15.26	0.548
Exterior inferior	294.60 ± 18.76	294.90 ± 17.40	0.564
Exterior temporal	297.55 ± 13.73	293.55 ± 13.21	0.936
RNLF thickness			
Average	111.90 ± 12.94	109.70 ± 9.42	0.621
Superior	129.80 ± 20.26	136.95 ± 21.22	0.507
Nasal	97 ± 17.61	85.50 ± 8.47	0.528
Inferior	148.90 ± 17.33	146.90 ± 19.2	0.916
Temporal	67.1 ± 6.99	65.75 ± 6.23	0.805
Optic disc parameters			
Disc area	2.37 ± 0.43	2.21 ± 0.44	0.223
Cup area	0.54 ± 0.29	0.60 ± 0.33	0.518
c/d horizontal	0.50 ± 0.14	0.51 ± 0.12	0.816
c/d vertical	0.44 ± 0.11	0.45 ± 0.11	0.813

**Table 2:** Correlation between refractive error and OCT parameters in amblyopic patients.

	*R* value	*P* value
Macular thickness		
Central zone	−0.26	0.914
Inner superior	0.80	0.738
Inner nasal	0.40	0.868
Inner inferior	−0.63	0.791
Inner temporal	−0.265	0.258
Exterior superior	0.95	0.689
Exterior nasal	−0.066	0.781
Exterior inferior	0.162	0.494
Exterior temporal	0.112	0.638
RNLF thickness		
Average	0.203	0.391
Superior	0.027	0.909
Nasal	0.419	0.066
Inferior	0.328	0.158
Temporal	−0.033	0.891
Optic disc parameters		
Disc area	0.463	0.960
Cup area	−0.320	0.168
c/d horizontal	−0.298	0.202
c/d vertical	−0.323	0.165

## References

[1] Wong A. M. F. (2012). New concepts concerning the neural mechanisms of amblyopia and their clinical implications. *Canadian Journal of Ophthalmology*.

[2] DeSantis D. (2014). Amblyopia. *Pediatric Clinics of North America*.

[3] Sapkota K. (2014). A retrospective analysis of children with anisometropic amblyopia in Nepal. *Strabismus*.

[4] Lee S. H., Chang J. W. (2014). The relationship between higher-order aberrations and amblyopia treatment in hyperopic anisometropic amblyopia. *Korean Journal of Ophthalmology*.

[5] Burke M. J., DeRespinis P. A., Medow N. B. (2013). Treatment of anisometropic amblyopia. *Journal of Pediatric Ophthalmology and Strabismus*.

[6] Chen B.-B., Song F.-W., Sun Z.-H., Yang Y. (2013). Anisometropia magnitude and visual deficits in previously untreated anisometropic amblyopia. *International Journal of Ophthalmology*.

[7] Mori T., Sugano Y., Maruko I., Sekiryu T. (2014). Subfoveal choroidal thickness and axial length in preschool children with hyperopic anisometropic amblyopia. *Current Eye Research*.

[8] Barnes G. R., Li X., Thompson B., Singh K. D., Dumoulin S. O., Hess R. F. (2010). Decreased gray matter concentration in the lateral geniculate nuclei in human amblyopes. *Investigative Ophthalmology and Visual Science*.

[9] Hess R. F., Thompson B., Gole G. A., Mullen K. T. (2010). The amblyopic deficit and its relationship to geniculo-cortical processing streams. *Journal of Neurophysiology*.

[10] Hess R. F., Thompson B., Gole G., Mullen K. T. (2009). Deficient responses from the lateral geniculate nucleus in humans with amblyopia. *European Journal of Neuroscience*.

[11] Liu H., Zhong L., Zhou X., Jin Q.-Z. (2010). Macular abnormality observed by optical coherence tomography in children with amblyopia failing to achieve normal visual acuity after long-term treatment. *Journal of Pediatric Ophthalmology and Strabismus*.

[12] Huynh S. C., Samarawickrama C., Wang X. Y. (2009). Macular and nerve fiber layer thickness in amblyopia: the Sydney Childhood Eye Study. *Ophthalmology*.

[13] Lempert P. (2008). Retinal area and optic disc rim area in amblyopic, fellow, and normal hyperopic eyes: a hypothesis for decreased acuity in amblyopia. *Ophthalmology*.

[14] Andalib D., Javadzadeh A., Nabai R., Amizadeh Y. (2013). Macular and retinal nerve fiber layer thickness in unilateral anisometropic or strabismic amblyopia. *Journal of Pediatric Ophthalmology and Strabismus*.

[15] Al-Haddad C. E., Mollayess G. M. E., Mahfoud Z. R., Jaafa D. F., Bashshur Z. F. (2013). Macular ultrastructural features in amblyopia using high-definition optical coherence tomography. *British Journal of Ophthalmology*.

[16] Alotaibi A. G., Al Enazi B. (2011). Unilateral amblyopia: optical coherence tomography findings. *Saudi Journal of Ophthalmology*.

[17] Ersan I., Zengin N., Bozkurt B., Özkagnici A. (2013). Evaluation of retinal nerve fiber layer thickness in patients with anisometropic and strabismic amblyopia using optical coherence tomography. *Journal of Pediatric Ophthalmology and Strabismus*.

[18] Birch E. E. (2013). Amblyopia and binocular vision. *Progress in Retinal and Eye Research*.

[19] El-Shamayleh Y., Kiorpes L., Kohn A., Movshon J. A. (2010). Visual motion processing by neurons in area MT of macaque monkeys with experimental amblyopia. *The Journal of Neuroscience*.

[20] Wang B. Z., Taranath D. (2012). A comparison between the amblyopic eye and normal fellow eye ocular architecture in children with hyperopic anisometropic amblyopia. *Journal of American Association for Pediatric Ophthalmology and Strabismus*.

[21] Dickmann A., Petroni S., Perrotta V. (2011). A morpho-functional study of amblyopic eyes with the use of optical coherence tomography and microperimetry. *Journal of AAPOS*.

[22] Xu J., Lu F., Liu W., Zhang F., Chen W., Chen J. (2013). Retinal nerve fibre layer thickness and macular thickness in patients with esotropic amblyopia. *Clinical and Experimental Optometry*.

[23] Tugcu B., Araz-Ersan B., Kilic M., Erdogan E. T., Yigit U., Karamursel S. (2013). The morpho-functional evaluation of retina in amblyopia. *Current Eye Research*.

[24] Firat P. G., Ozsoy E., Demirel S., Cumurcu T., Gunduz A. (2013). Evaluation of peripapillary retinal nerve fiber layer, macula and ganglion cell thickness in amblyopia using spectral optical coherence tomography. *International Journal of Ophthalmology*.

[25] Miki A., Shirakashi M., Yaoeda K. (2010). Retinal nerve fiber layer thickness in recovered and persistent amblyopia. *Clinical Ophthalmology*.

[26] Yalcin E., Balci O. (2014). Peripapillary retinal nerve fiber layer and foveal thickness in hypermetropic anisometropic amblyopia. *Clinical Ophthalmology*.

[27] Kim Y. W., Kim S.-J., Yu Y. S. (2013). Spectral-domain optical coherence tomography analysis in deprivational amblyopia: a pilot study with unilateral pediatric cataract patients. *Graefe's Archive for Clinical and Experimental Ophthalmology*.

[28] Walker R. A., Rubab S., Voll A. R. L., Erraguntla V., Murphy P. H. (2011). Macular and peripapillary retinal nerve fibre layer thickness in adults with amblyopia. *Canadian Journal of Ophthalmology*.

[29] Kantarci F. A., Tatar M. G., Uslu H. (2015). Choroidal and peripapillary retinal nerve fiber layer thickness in adults with anisometropic amblyopia. *European Journal of Ophthalmology*.

[30] Araki S., Miki A., Yamashita T. (2014). A comparison between amblyopic and fellow eyes in unilateral amblyopia using spectral-domain optical coherence tomography. *Clinical Ophthalmology*.

